# Design and Fabrication of Low-Cost Microfluidic Chips and Microfluidic Routing System for Reconfigurable Multi-(Organ-on-a-Chip) Assembly

**DOI:** 10.3390/mi12121542

**Published:** 2021-12-11

**Authors:** Sadeq Abu-Dawas, Hawra Alawami, Mohammed Zourob, Qasem Ramadan

**Affiliations:** College of Science and General Studies, Alfaisal University, Riyadh 11533, Saudi Arabia; saabudawas@alfaisal.edu (S.A.-D.); hfalawami@alfaisal.edu (H.A.)

**Keywords:** microfluidics, organ-on-a-chip, assembly, configurable

## Abstract

A low-cost, versatile, and reconfigurable fluidic routing system and chip assembly have been fabricated and tested. The platform and its accessories were fabricated in-house without the need for costly and specialized equipment nor specific expertise. An agarose-based artificial membrane was integrated into the chips and employed to test the chip-to-chip communication in various configurations. Various chip assemblies were constructed and tested which demonstrate the versatile utility of the fluidic routing system that enables the custom design of the chip-to-chip communication and the possibility of fitting a variety of (organ-on-a-chip)-based biological models with multicell architectures. The reconfigurable chip assembly would enable selective linking/isolating the desired chip/compartment, hence allowing the study of the contribution of specific cell/tissue within the in vitro models.

## 1. Introduction

Recent advances in tissue engineering have shown great potential in producing human tissue/organs in vitro that could be used as animal alternative models. Tissue engineering involves the combination of living cells with natural or synthetic support to develop 3D living structures that are structurally and functionally equal to living tissues [1]. The structure of living organs is very complex and it is not possible to exactly mimic with currently available technology. Thus, the aim of in vitro modeling is not to replicate the native tissue/organ, but to recapitulate the main structural components and conditions that enable capturing key physiological parameters which are relevant to particular patho-/physiological conditions or enable studying the effect of the tissue on exogenous stimuli (pharmacokinetics) or the impact of these exogenous substances on the body (pharmacodynamics).

In vivo, organs are connected via networks of blood and lymphatic vessels and communicate via signaling molecules and exosomes, and are controlled by the autonomic nervous system and the endocrine system. Linking different organ/tissue modules in vitro can greatly affect their functions and effectiveness. This cellular organization reflects the tissue function and determines the cell–cell communication within an individual organ, and with distant organs, through secreting various soluble factors and extracellular vesicles [2].

**Figure 1:**
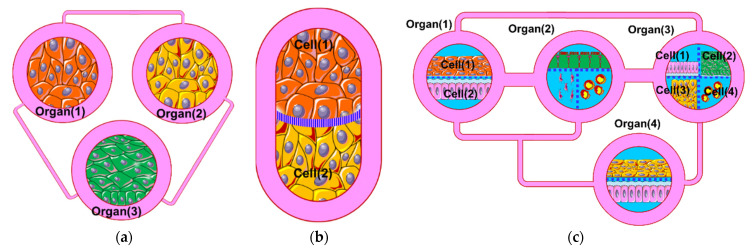
Three strategies of mOOC perfusion systems. (**a**) Individual tissue models (OOCs) linked together through microchannels or tubing. (**b**) Two (or more) tissue models (OOCs) interfaced through a thin, porous barrier (e.g., porous membrane). (**c**) Several organ models linked together through tubing. Each organ model comprises two or more cell types interfaced through thin, porous barriers.

To create viable in vivo-like in vitro models with different types of cells, cells originated from an individual organ need to be cocultured in close proximity within an environment that ensures a continuous supply of nutrients through artificial or biomimetic vasculature, a minimum level of cell–cell crosstalk and maintain continuous paracrine signaling. Organ-on-a-chip devices promote organotypic structures by organizing various types of cells with the appropriate tissue architecture and precise cellular positioning mimicking cell sheets, ducts, or more complex cellular organizations [3,4]. On the other hand, in vitro integration of multiorgans within one enclosed system requires a different type of engineered system that depends on the type and number of the integrated organs, and a sophisticated perfusion circuit that recapitulates the physiological flows and maintains long-term cell manipulation and signal capturing without compromising the cell viability. Increasing the number of integrated organs would increase the biological complexity; hence it requires yet more complex fluidic architecture. For instance, a “human-on-a-chip” device would include many tissue/organ models; therefore, it requires a sophisticated perfusion circuit (i.e., artificial circulation system and other connection ducts) to ensure mimicking a long-term organ-organ interaction in health and disease states (Figure 1a).

OOCs are characterized by the dynamic long-term coculture of heterogenous cells arranged in precise geometries of perfusion microfluidic chips that lead to a complex interaction between cells and the microenvironment. Different types of cells may require different nutrients and growth factors and may not be compatible with coculture before differentiation/maturation. In addition, the cellular architecture, including organization, polarity, and interface, varies in different organs. During the last two decades, a plethora of studies have been published with the vast majority of these studies focusing on constructing the biological models, either as a single organ model or multiorgan models, utilizing simple engineered rapid-prototyped devices with only a few studies on engineering devices which are capable of hosting complex organotypic in vitro models.

To realize a highly integrated biological model with multitissue types or multi organs, three options can be adopted:

(a) Creating several simple microfluidic chambers/chips, within each, a cell monoculture is hosted, which is connected either through microchannels or tubing [5,6,7,8,9,10] (Figure 1a). This design enables an easy and simple connection of many organ modules using external channels/tubing with the possibility of selectively switching the flow into the individual chip (ON/OFF) or directing the flow using external fluidic valves. In addition, modules can be connected/disconnected at the desired time (e.g., after cell differentiation). However, the organization of cell types in this design does not mimic that in vivo, where the cell–cell interfacing distance (i.e., the channel/tubing) between the various types of cells is relatively large. This design is suitable to link cells/tissues, which are separated a distance between each other such as heart–liver, intestine–liver, etc., but do not provide a physiologically relevant cell–cell interfacing when used to model the heterogeneous cellular structure of an individual organ, such as the liver which contains several cell types (hepatocytes, hepatic stellate cells, sinusoidal endothelial cells, and Kupffer cells), or interfacing the parenchyma and nonparenchymal cells or immune cells where cells are very close to each other. Zhang et al., 2017 [11] reported an integrated modular sensing platform through a fluidics-routing breadboard, which operates multiorgan-on-a-chip units. The system comprises built-in pneumatic valves, micro bioreactors for housing organoids, and biochemical, physical, and optical sensors. The individual modules are selectively connected/disconnected with Teflon tubes. The system was tested for long-term monitoring of drug-induced organ toxicity in hepatic and cardiac organoids. Few commercial platforms became available. For example, TissUse GmbH (Berlin, Germany) introduced a variety of HUMIMIC Chips, such as HUMIMIC Chip2, HUMIMIC Chip3, HUMIMIC Chip4, and HUMIMIC Chip XX/XY, for different in vitro modeling purposes, which enable the integration of 2, 3, and 4 organs [12].
(b) Integration of multiorgans on a single chip. In this design, the chip comprises multiple fluidic chambers, with each compartment hosting a specific organ, tissue, or homogeneous single cell type culture (Figure 1b). Each chamber may host one or two cell types within single or multiple compartments (e.g., two stacked compartments separated by a porous membrane) that emulate a specific organ. The fluidic chambers are linked either by physical or biological barriers that ensure continuous organ-organ crosstalk [13,14,15,16,17,18,19,20]. This design facilitates efficient cell–cell and tissue-tissue interaction between heterogeneous cell cultures, and allows spatial organization of different types of cells/tissue in 2D and 3D architectures with physiologically relevant orientation. In addition, the fluidic architecture allows the recruitment of circulating immune cells with a high spatiotemporal resolution of cell–cell interaction [3,13,15]. However, the limited diffusion through the physical/biological barriers limits the number of fluidic chambers, and hence, the number of cell types within the system. Therefore, the biological complexity of the system would require a more sophisticated design of the fluidic chip to enable efficient fluidic exchange or diffusion through the multichamber system.
(c) Simple OOC chips within each 2–3 fluidic compartments can be integrated by externally linking them through tubing and valves (Figure 1c). This design allows for integrating relatively complex individual organ models (e.g., liver) within individual chips and integrating several organ modules in one perfusion system (e.g., intestine, liver, skin, heart, and kidney).

In this paper, we adopted the latter approach to develop a low-cost modular microfluidic platform for cell coculture that enables a flexible design of in vitro cellular architecture with the ability to modulate/reconfigure the structure and the flow direction prior to and during the experiments. The perfusion system comprises two major components: (1) a 3D microfluidic chip which comprises two vertically stacked fluidic compartments separated by a porous membrane; and (2) a fluidic routing unit that is capable of accommodating 6 chips with reconfigurable chip assembly.

## 2. Fabrication

### 2.1. Fabrication of the 3D Microfluidic Chip

**Figure 2:**
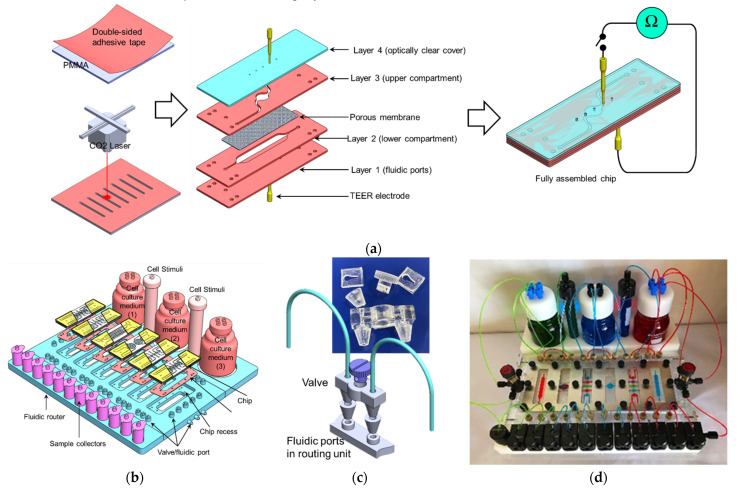

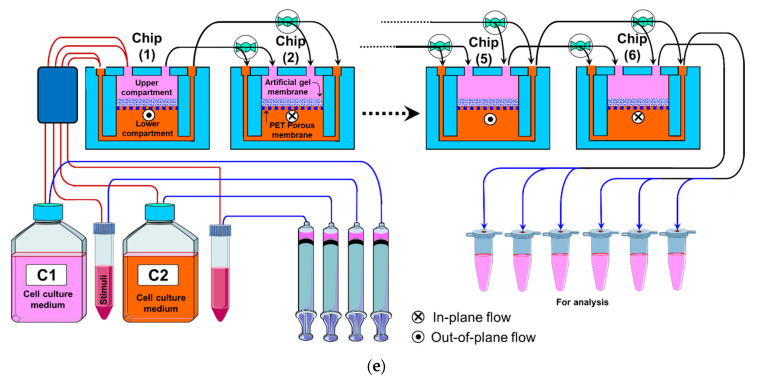
(**a**) Fabrication of the microfluidic chip. The chip was assembled of four PMMA sheets with microfluidic features created using the laser cutting technique. A porous membrane separates the two (upper and lower) microfluidic compartments. Gold electrodes with a diameter of 500 µm were inserted into the top and bottom cover to access the upper and lower compartment for TEER measurements. (**b**) 3D drawing of the perfusion system with its major components. (**c**) 3D-printed micro valve which was used to regulate the flow within the chip assembly (**d**) An image of the fully assembled perfusion system (colored liquid was used for better visualization). (**e**) The microfluidic chip assembly with an example of possible chip-chip connecting.

The microfluidic chip was fabricated of poly(methyl methacrylate) (PMMA) using laser cutting technique. The chip was first drawn using AutoCAD software (Autodesk, San Rafael, CA, USA). Double-sided adhesive tape (3M, Saint Paul, MN, USA) was laminated on a 1 mm thick PMMA sheet and then the designed features were cut using the CO_2_ laser cutter (Beambox, Flux, Taipei, Taiwan). To create a fully sealed microfluidic chip, four PMMA layers were assembled with two patterned layers which form the upper and lower fluidic compartments, and one layer with fluidic ports and one layer as a cover (Figure 2a). A polyethylene terephthalate (PET) membrane (Millipore, Burlington, MA, USA), with a pore size of 0.4 μm and pore density of 4 × 10^6^ pores/cm^2^ was sandwiched between the two patterned PMMA sheets. To realize a complex fluidic routing structure, six chips were included in the fluidic assembly. The individual chip comprises two vertically stacked compartments sandwiching a porous membrane. The lower compartment has a rectangular shape with a length and width of 40 mm and 4 mm, respectively, whereas the upper compartment has a circular shape with a diameter of 4 mm (Figure 2a). The design of the chip with a larger lower compartment ensures 100% intersection of the fluid within the upper compartment with that in the lower compartment, which is necessary in case of epithelial cell culturing in the upper side. On the other hand, not all the fluid within the lower compartment intersects with the upper compartment as the lower compartment is used mainly as a feeding channel. In the current study, the upper compartment is designed with a circular shape which may result in inhomogeneous shear stress over its surface at certain flow rates. However, when the shear stress is crucial, such as in the case of endothelial cell layers, a straight narrow channel can be employed. The external dimensions of the microfluidic chips had a length of 75 mm, width of 25 mm, and depth of 1 mm.

To monitor epithelial cell integrity, gold wires with a diameter of 0.5 mm (World Precision Instruments INC., Sarasota, FL, USA) were inserted into the upper and lower compartments by punching holes in the top and bottom covers (Figure 2a). In each culture chamber, a gold wire was inserted above and beneath the PET membrane. These electrode wires were used to measure the transepithelial electrical resistance (TEER) of the cell sheets, which is considered an indicator of the cell layer integrity. These electrodes enable measuring TEER through the course of cell culture and assessment of cell layer confluence as well as detection of the impact of chemical/physical stimuli on the intercellular tight junction (TJ) integrity.

### 2.2. Fabrication of the Fluidic Routing Unit

The function of the fluidic routing unit, which accommodates 6 interconnected microfluidic chips, is to enable the reconfigurable chip-to-chip fluidic connection (Figure 2b). By employing a set of integrated valves (Figure 2b inset), every individual chip can be connected to the next chip with two different options: (1) the upper compartment of Chip(1) is connected to the upper compartment of Chip(2) or (2) the upper compartment of Chip(1) is connected to the lower compartment of Chip(2). The flow between the connected compartments can be switched ON/OFF using the corresponding valve. The fluidic router was fabricated using a laser cutter, micro-milling, PMMA machining, and 3D printing techniques using PMMA which contain the following components (Figure 2b,c):

(1) Six recesses with dimensions that enable accommodating the chip and channels network (with an individual channel diameter of 500 µm). Within each recess, a set of L-shaped channels were made to connect the chip ports to the external tubing and the injection syringes.
(2) A set of 3D-printed fluidic valves: The valve was designed using SolidWorks software (Dassault Systèmes, Vélizy-Villacoublay, France) and printed using an ASIGA MAX UV 3D printer (NSW, Australia). The valve comprises a rotating cylindrical rod with a 500 µm perpendicular through hole. When the hole is aligned with the fluidic channel, it allows the fluid to pass through (OPEN). Rotating the rod 90°, the fluidic channel and the valve’s hole will be perpendicular to each other, which would block the flow through the channel (CLOSE) (Figure 2c).
(3) The fluidic router also allows for connecting the chips to cell culture media and stimuli reservoirs as shown in Figure 2b,d,e. Pinch valves were used to control the flow from the reservoirs.

## 3. Materials and Methods

### 3.1. The Perfusion Setup

The main aim of the study was to demonstrate a reconfigurable perfusion system that is capable of hosting mOOCs for the desired time with the flexibility of switching and directing the flow within an individual chip and within the chip assembly. Figure 2b,c show the six chips assembled in the fluidic routing unit. The chips were inserted in the chip recess with the fluidic ports aligned to those in the recess. A silicone sheet with a thickness of 200 µm and through-holes with diameters of 1 mm patterned corresponding to the fluidic ports on the chip were placed beneath the chip to ensure acceptable fluidic sealing. The fluidic inlets of the culture chambers were connected to the culture media reservoir through a set of PEEK tubing with an inner diameter of 0.5 mm. The flow from the reservoir through the chips was driven by a set of programmable syringe pumps (KDScientific, Holliston, MA, USA). To maintain a steady culture environment (i.e., temperature and O_2_ supply) during experiments, the entire perfusion system, except the syringe pumps, were kept in the CO_2_ incubator. Positive pressure was applied onto the syringes to maintain continuous perfusion of cell culture media through the cell culture chambers at a flow rate of 10–20 nL/s. To characterize the perfusion system and its ability to circulate the fluid in various chip assembly setups, chips were connected in various chip-chip connections (Figure 2e) and the flow was diverted using two types of valves: (1) 2 to 1 L-shaped valve which switches the flow between two routes and (2) a V-shaped pinch valve which either allows or stops the flow in a short Tygon tubing connecting two fluidic ports by squeezing/releasing (OFF/ON) the tube. Dyed water (red, green, blue) was used to visualize the flow redirection in the system (Figure 2d).

### 3.2. TEER Measurements

The gold wires were sterilized with isopropanol (IPA) followed by PBS, dried and irradiated with UV light prior to inserting into the chip as shown in in Figure 2a. The other ends of the wires were then connected to a digital multimeter (BK Precision, Yorba Linda, CA, USA) to measure the TEER. One chip without an artificial membrane/cells was used as a control. TEER measurements were obtained at regular intervals with each data point being a mean of three measurements. The contribution of the gel-coated membrane or cells was estimated by subtracting the electrical resistance of the porous membranes without gel/cells from the total measured values, and normalized by multiplying the measured electrical resistance by the total surface area of the gel/cells to calculate TEER in Ω·cm^2^.

### 3.3. Trans-Membrane Permeability

The permeability of FITC-dextran (4K Da) was measured through artificial membranes/cell layers. The artificial membrane was constructed by coating the PET porous membrane with agarose gel to emulate the transepithelial permeability. One gram of agarose powder was dissolved in 100 mL of TAE buffer and heated up to 80 °C for 5 min. Then, ~2 µL, ~4 µL, and ~6 µL of dissolved agarose gel was placed on top of the PET membrane within the upper compartment of three different chips to form the gel-based artificial membrane with a thicknesses of 50 µm, 100 µm, and 150 µm, respectively. The upper and lower compartments were filled with a DMEM cell culture medium using syringe pumps and placed in the CO_2_ incubator for at least 1 h. Ten μL of the FITC-dextran solution with a concentration of 10 μg/mL was injected into the upper/lower compartment of the chip (or the first chip) and the transmembrane flux of the tracer molecule was quantified by serially sampling fluid from the acceptor compartment of the last chip, according to the chip assembly, at different time intervals using an Enspire fluorescent plate reader (Perkin Elmer, Richmond, CA, USA). The associated TEER values with sampling were also measured.

### 3.4. Cell Culture

#### 3.4.1. Immune Cells

An immortalized immature dendritic cell line, JAWS II (ATCC, Manassas, VA, USA), was used as a model of immune responsive cells. Cells were cultured in a complete culture medium consisting of IMDM with 10% FCS, 4 mM L-glutamine, 10 U/mL penicillin and 100 μg/mL streptomycin, 0.5 mM 2-ME, and 1 mM sodium pyruvate. Nonadherent cells were transferred to a centrifuge tube and the attached cells were treated with 0.25% trypsin 0.03% EDTA (Gibco) at 37 °C for 5 min and transferred to the centrifuge tube. Then the two populations were transferred to a new flask. Before cell inoculation, the cell culture chips were washed thoroughly with 70% ethanol, rinsed with distilled water, exposed to UV light for at least 30 min, and incubated with cell culture media overnight in a cell culture incubator (37 °C with humidified atmosphere and 5% CO_2_). JAWS II cells were inoculated into the lower compartment via the corresponding fluidic inlet and cell-free media was injected into the upper compartment. Fresh culture media was perfused into the chip through the syringe pump at a constant flow rate of 20 nL/s. To quantify cell viability, cells were mixed with trypan blue at a 1:1 ratio and counted for living and dead cells using an automated cell counter (Millipore, Burlington, MA, USA).

#### 3.4.2. Epithelial Cells

Caco-2 cells (CLS Cell Lines Service GmbH, Eppelheim, Germany) were also used to construct an epithelium model on a chip. Before cell seeding, the chips were flushed with 70% ethanol and dried and exposed to UV irradiation for 30 min. The chips were then filled with culture medium, which is composed of Dulbecco’s modified Eagle’s medium (DMEM) with 4.5 mg/mL glucose, 50 U/mL penicillin, 50 U/mL streptomycin, 4 mmol/L glutamine, 25 mmol/L HEPES, and 10% fetal bovine, and preincubated overnight. The cells from 10–15 passages were inoculated into the upper compartment of the chip at a concentration of ~105 cells/ cm^2^ using a syringe pump and maintained in the culture medium. After cell seeding, the perfusion flows were stopped to allow cell attachment onto the membrane. After 5 h, the culture media were allowed to flow using the syringe pump at a flow rate of 10–20 nL/s. The microfluidic culture was maintained at 37 °C in a humidified incubator with 5% CO_2_.

#### 3.4.3. Cell Viability

A mixture of calcein-AM (2 μM) and ethidium homodimer-1 (4 μM) diluted in D-PBS was introduced into the upper compartment and incubated for 10 min. Then the cell viability was examined by a fluorescent microscope. Cell viability was calculated as the percentage of calcein AM-labeled cells. Images were taken from different locations and the percentage value was averaged. Data were represented as mean ± SD.

## 4. Results and Discussion

### 4.1. Fluidic Characterization

**Figure 3:**
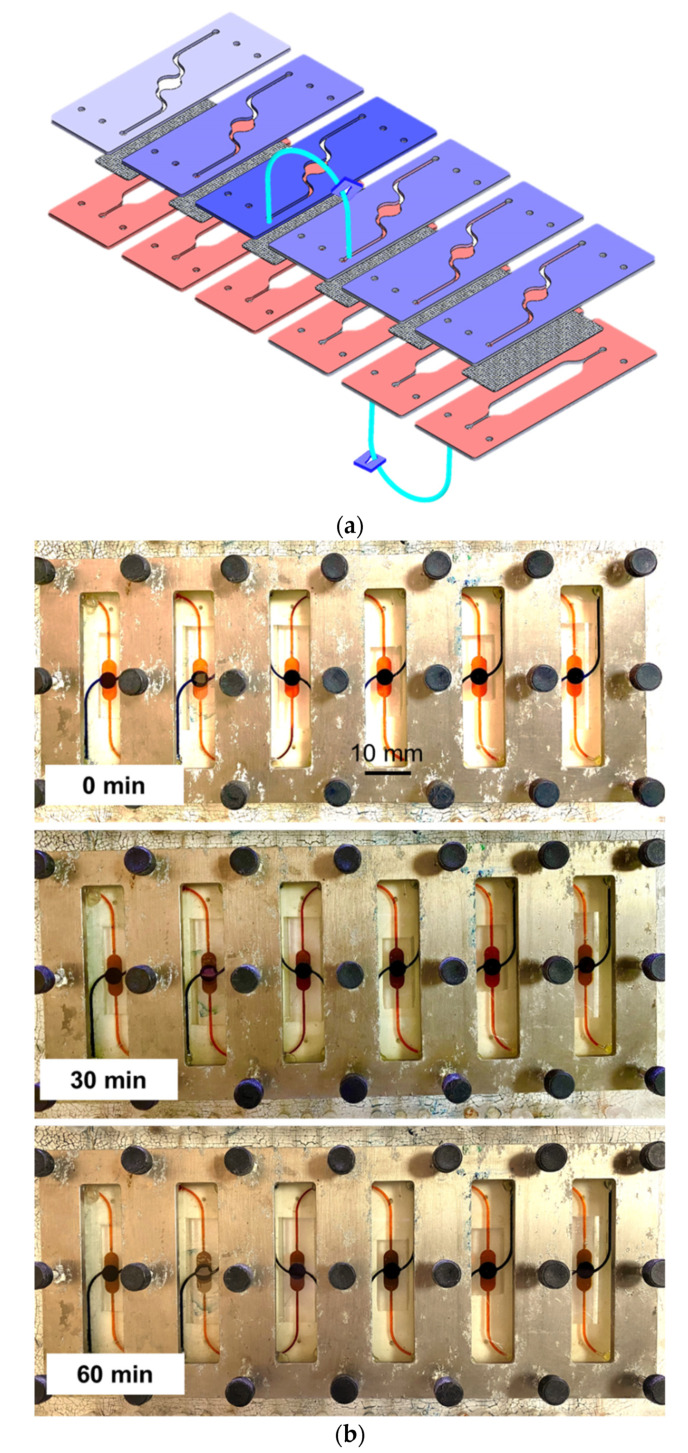
Fluidic routing through chip assembly. (**a**) Connection of the chip assembly, (**b**) images showing the color mixing due to the fluidic transfer through chips.

The fluidic routing through multicompartment chip assembly was visualized by injecting colored water (red, green, and blue) into the chips and color mixing, which indicates the cross-compartment fluidic mixing and transfer. Figure 3a shows a selected chip assembly through which the colored water flows and Figure 3b shows detailed images of the compartments at the time of injection, after 30 min and after 60 min of injection. The fluid routes through the different chips, bypassing the porous membrane where porosity determines the speed of the fluid exchange as well as the concentration difference across the compartments above and under the membrane.

### 4.2. Tranepithelial Electrical Resistance (TEER) Characterization

**Figure 4:**
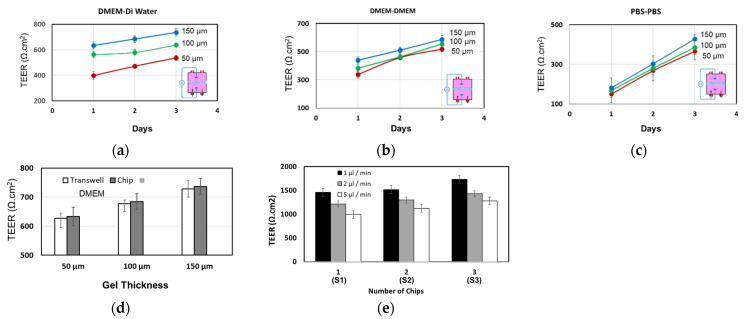
(**a**–**c**) The TEER values of the artificial agarose-coated membrane with three different thicknesses (50 µm, 100 µm and 150 µm) measured in individual microfluidic chips at a flow rate of 5 µL/min. (**d**) The measured TEER values within the chip were higher than those in the transwell with the same gel thicknesses. Flow rate in the chip was 5 µL/min. (**e**) TEER values measured across two membranes within two chips which are connected through the lower compartments.

To characterize the integrated TEER electrodes, artificial membranes were built on top of the PET membrane, which were made of agarose gel with thicknesses of 50 µm, 100 µm, and 150 µm. TEER electrodes were inserted as shown in Figure 2a and the TEER values were recorded at different time intervals. Three different solutions within the upper/lower compartments were used: DMEM/DMEM, PBS/PBS, and DMEM/distilled water. The conductivities of DMEM, PBS, and DI water were 14 mS/cm, 17 mS/cm, and 0.0017 mS/cm, respectively. Higher TEER values were measured for the thicker, gel-coated membrane and a slight increase of the TEER was also observed with time (Figure 4). This can be attributed to the possible change of gel-coated membrane porosity and structure during the course of the experiment when it is suspended in the DMEM or PBS solutions. It should be noted that the agarose gel was used only to demonstrate the structure of the chip and chip assembly and several other materials can be used to construct the artificial membrane, such as the lipid mixture used in the artificial membrane permeability assay (PAMPA) [21,22].

The TEER values across the artificial membrane were observed to be the highest when DMEM (upper) and DI water (lower) was used, due to the low conductivity of water compared to DMEM and PBS. On the other hand, TEER values were the lowest when PBS (upper) and PBS (lower) were used, due to the high conductivity of the PBS. Higher TEER values were also noticed in microfluidic chips compared to those in transwell for the same membrane thickness (Figure 4d), due to the presence of fluid flow in the chip. Two chips were fluidically linked through the lower compartment, the TEER electrodes were inserted in the upper compartment of the two electrodes, and TEER was measured across the two membranes as shown in Figure 4e. The measured TEER across the two membranes was higher than those across the membrane at the same membrane’s thickness.

### 4.3. Permeability Characterization

**Figure 5:**
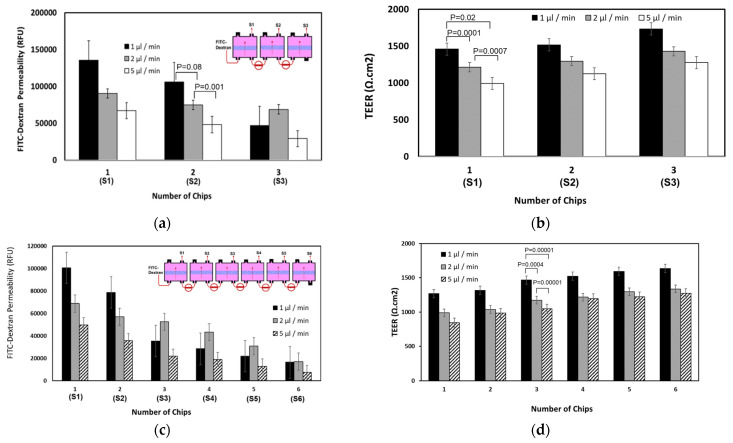
(**a**) The permeability of FITC-dextran through 3 chips linked to each other in a series through the lower (basolateral) compartments. The permeability was detected by measuring the intensity of FITC in the upper compartments. S indicates the sample collected from the corresponding upper compartment. (**b**) The corresponding TEER values. (**c**) The permeability of FITC-dextran through 6 chips. (**d**) The corresponding TEER values. Student t-test was used for statistical analysis. *p* < 0.05 was considered significant.

The permeability of FITC-dextran (4K Da) was measured through the artificial agarose-coated membranes using various configurations of the chip assembly. For example, three or six identical chips were linked together through the lower compartments such that all the lower compartments became merged as a single compartment, and the liquid flowed steadily through one long channel (Figure 5). FITC-dextran solution with a concentration of 10 μg/mL was injected into the lower compartment of the first chip and allowed to flow downstream through the other chips at selected flow rates (i.e., 1 μL/min, 2 μL/min, and 5 μL/min). Samples were collected from the upper compartment of each chip after 6 h and the fluorescent intensities of each sample were measured. The associated TEER value was also measured at sampling time. As shown in Figure 5a,c, the FITC intensity steadily decreases downstream, being highest in the first chip (upper compartment) and lowest in the last chip (upper compartment). The steady decline of dextran concentration through the chips indicates a steady mass transfer and acceptable chip-chip communication through the assembly. FITC-dextran loss in the agarose-coated membrane may also slightly contribute to the decrease of the downstream concentration of dextran. No significant TEER modulation was observed through the chip assembly, but a slight decrease of TEER was observed with increase in the flow rate (Figure 5b,d).

These setups can be exploited to measure the permeability of desired substances from the lower channel (emulating the bloodstream) into various tissues (in the upper compartments). These tissues can be identical so that parallel identical experiments can be run on different tissues/organs to investigate the possible distribution of substances to different tissues. The setups can be useful to demonstrate various biological models and experiments for pharmacokinetic applications.

### 4.4. Epithelial Cell Culture Testing

**Figure 6:**
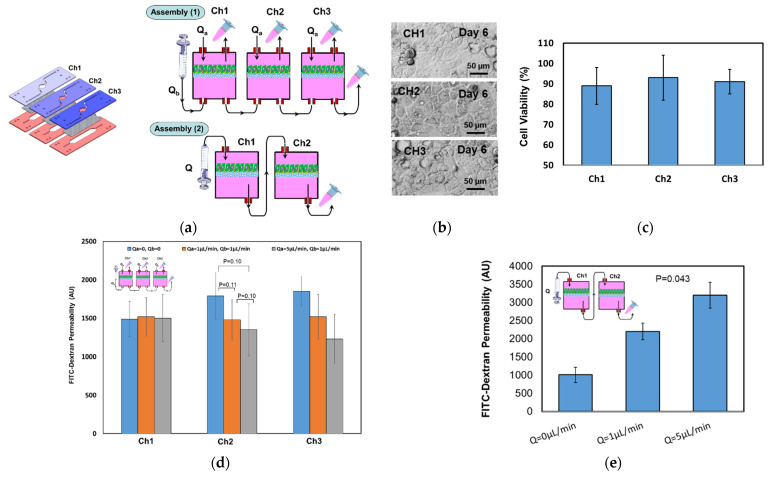

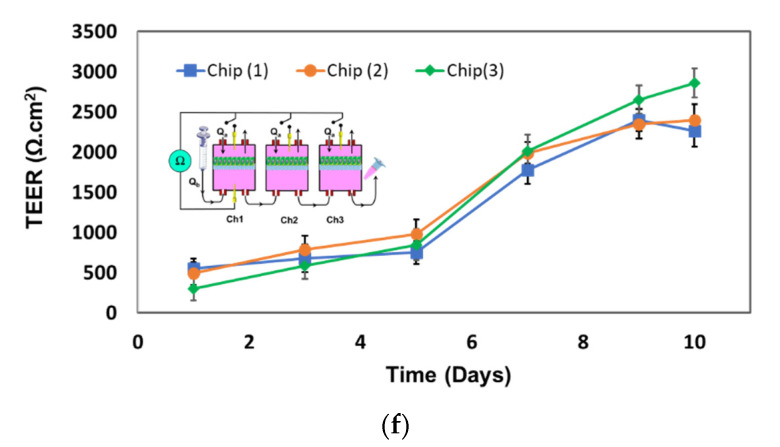
(**a**) Three chip assembly of each comprised one basolateral and detailed view of the through compartment fluidic connection. (**b**) Caco-2 cells formed a fully confluent layer on top of a PET membrane. The images were taken from the apical side of the chips. (**c**) Cell viability (at day 6) in the various compartments all showed acceptable viability. (**d**) Fluorescent intensity due to the diffusion of FITC-dextran 4 kDa through the caco-2 cell layer(s) in three-chip assembly connected through the basolateral compartments. (**d**) Two-chips assembly with an apical-basolateral connection. (**e**) The measured TEER values of the caco-2 monolayer against time in the three-chip assembly. The TEER increases with time due to the cell’s continuous growth and covering of the membrane surface. (**f**) The TEER was measured in individual chips. A Student t-test was used for statistical analysis. *p* < 0.05 was considered significant.

Fully confluent layers of caco-2 cells were created in a three-chip assembly (Figure 6a,b). The cell culture was maintained in the CO_2_ incubator and the cell growth was monitored regularly under a microscope. Figure 6b shows three bright field images taken at day 6 of cell culture from the apical compartments, and Figure 6c shows acceptable cell viability (> 89%) through the different chips. For testing the chip-to-chip communication, two different fluidic connections were utilized. In the first assembly (Assembly (1)), the lower compartments of the three chips were connected in a series, whereas the upper compartments were kept separated (Figure 6a). The second assembly (Assembly (2)) comprised two chips with the lower compartment of chip (1) connected to the upper compartment of chip 2 (Figure 6a). The two-chip assembly has only one inlet and one outlet. FITC-dextran solution was initially injected either in the first lower compartment of the assembly (1) or the first upper compartment of the assembly (2). Then, the infiltrates were collected from the upper compartments (Assembly (1)) and the lower compartment (Assembly (2)), and the fluorescent intensity (FI) was measured using a fluorescent plate reader. Figure 6d shows the FI, which is correlated to the permeability through the upper compartments of the three chips in static condition and at two different apical/basolateral flow rates (Qa = 1 µL/min, Qb = 1 µL/min, Qa = 5 µL/min, and Qb = 1 µL/min). The permeability slightly decreased when the basolateral flow rate increased, whereas the FI values within the three apical compartments were similar in the static condition. However, in the first chip, no change in the permeability was observed at different flow rates. This can be due to the location of the chip being close to the fluidic inlet, which leads to more markers flowing downstream toward the second and third chips due to the apical flow (Q_a_). In Assembly (2), all four compartments of the two chips were linked together and the liquid flowed in one direction through two membranes. The flow direction was from the apical to basolateral to mimic the direction of molecule transport (absorption through the small intestine). The FI increases with the flow rate as shown in Figure 6e, as the higher flow rate promotes more molecules to pass through the membrane. The upper compartments in the three chips shown in Figure 6 were identically seeded with caco-2 cells under the same conditions. In addition, TEER was monitored in individual chips in the three-chip assembly during the course of cell culture until a confluent layer was observed. TEER increased with time, as shown in Figure 6f, which indicates a successful cell culture within the chip assembly. This setup provides a tool for versatile experimental conditions to be exploited. For example, caco-2 monoculture can be studied in parallel and compared with a coculture of caco-2 cells and mucus-producing cells (goblet cells), or by adding artificial mucus to investigate the role of mucus in drug pharmacokinetics and as an immune component.

## 5. Conclusions

In this paper, we have presented the design, fabrication, and characterization of a low-cost microfluidic chip and a fluidic routing unit that enables custom and reconfigurable chip assembly. The platform and its accessories were fabricated in-house without the need for costly specialized equipment nor specific expertise. The various chip assemblies shown in the above experiments demonstrate the versatile utility of the fluidic routing system which enables custom design of the chip-to-chip communication and the possibility of fitting a variety of biological models with multicell architectures. The chip assembly was demonstrated for up to six chips, but a larger number of chips can also be similarly assembled.

## Data Availability

The data that support the findings of this study are available from the corresponding author Qasem Ramadan, upon request.
